# Pain expectation and avoidance in the social context: an electrophysiological study

**DOI:** 10.1186/s12576-021-00813-1

**Published:** 2021-09-06

**Authors:** Alessandro Piedimonte, Denisa Adina Zamfira, Giulia Guerra, Sergio Vighetti, Elisa Carlino

**Affiliations:** 1 Department of Neuroscience “Rita Levi Montalcini”, University of Turin, 10125 Turin, TO Italy; 2 Carlo Molo Foundation, Turin, Italy

**Keywords:** Pain, Electrophysiology, Contingent negative variation, Motor responses, Social learning

## Abstract

Contingent negative variation (CNV) is an informative electrophysiological measure of pain anticipation showing higher amplitudes when highly painful stimulation is expected while presenting lower amplitudes when low painful stimulation is expected. Two groups of participants were recruited: one group expected and received an electrical stimulation of different intensities while being alone in the room (i.e. without social context), while a second group performed the same experiment with an observer in the room (i.e. with social context). Lower pain ratings and slower reaction times were observed in the group with social context and these results were accompanied in this group by a lower amplitude in the early component of the CNV as well as a lower amplitude of the later component of the wave. These results show that CNV can be considered a precise measure of central elaboration of pain anticipation explaining both its perceptual and motor components.

## Background

Experimental as well as clinical evidences have shown that pain is a complex experience modulated by different factors [1–3]. These factors include cognitive variables, such as expectations and memories about previous painful experiences, and emotional variables, such as anxiety [2, 4]. For instance, studies on placebo hypoalgesia and nocebo hyperalgesia have shown that expectations about an incoming pain stimulation can change the subjective experience of pain. This subjective change occurs along with different objective pain processes, measured by means of electrophysiological potentials, such as evoked potentials that occur after a painful experience, or event-related potentials that anticipate a painful event [5, 6].

Two further factors that affect pain processes are the motor preparation required to avoid or stop a painful experience and the social context in which pain is experienced. Motor preparation to avoid a potential threat represents a fundamental component in pain processing, as documented by fMRI studies indicating that areas with a crucial role in motivated voluntary action, such as the orbitofrontal and cingulate cortex, are highly active when movements are performed while receiving painful stimulations compared to actions performed while not receiving any stimulation [7, 8]. Crucially, as recently shown in different studies on brain activity using electrophysiological measures (EEG), a single slow cortical potential, the contingent negative variation (CNV), seems to well dissect sensory and motor components of pain expectation [6, 9]. CNV is not a unique wave and is traditionally divided in two different components (namely the early and late CNV) [9–12] with different neural generators that include the prefrontal cortex, anterior cingulate cortex, premotor cortex and supplementary motor area (early component) and basal ganglia, prefrontal and premotor cortices and dorsal anterior cingulate cortex (late component) [13–16]. While the late CNV is detected just prior to the onset of the imperative stimulus and is clearly related to motor anticipation and preparation [17], the early component is more complex. It is thought to be related to initial attention to the warning stimulus, to the cognitive effort to respond to the imperative stimulus and to motivation to respond [18]. Studies on the electrophysiology of pain anticipation conducted with placebo and nocebo paradigms showed that the early phase of the CNV seems to be more related to changes in pain expectancy, so that its amplitude increases when participants anticipate high-intensity stimulations. On the other hand, the late phase of the CNV seems to be more related to pain avoidance, so that its amplitude increases when participants show faster reaction times, typically in correspondence with high-intensity stimulation [6].

Finally, social and interpersonal characteristics of the context in which pain is perceived crucially affect pain experience. Indeed, using a cold-pressor task, it has been shown that participants who received the experimental pain in the presence of another person experimented a positive increase in pain threshold as well as in pain tolerance (i.e. a general pain perception reduction) [19]. While the mere presence of another person during pain perception has been less studied, different studies focused on the effects of touch and empathy on pain perception in a social environment. Indeed, it has been shown that women who were administered tonic heat stimuli, perceived less pain while their partners touched their hands compared to when their partners just watched them. Furthermore, authors of this study found a significant impact of relationship quality and empathy on pain reduction so that women who had a better relationship with their partners but also women who had a partner with high empathy scores perceived a higher pain reduction [20]. Interestingly, the same authors later found that hand holding during pain perception increases brain to brain coupling in the alpha band (8–12 Hz) and this increase is correlated with pain perception reduction but also with empathy scores of the person who is holding the hand of the pain receiver [21]. Furthermore, it has been recently discovered that the mere presence of another person is sufficient to reduce autonomic responses to aversive events, such as human screams, measured by a reduction in skin conductance responses [22].

On the basis of this literature, the aim of the current study is to directly investigate pain anticipation and perception as well as the motor responses elicited to stop noxious stimuli in a social context by means of both behavioural and electrophysiological measures. To achieve this goal, two groups of participants were recruited for the study: one group expected and received a low-intensity or high-intensity electrical stimulation without any other participant in the room (i.e. without social context) while the other group followed the same paradigm with another participant in the room (i.e. with social context). Participants had to stop the stimulation as soon as possible and rate its intensity on a numerical rating scale while EEG was simultaneously recorded. Reaction times, rating scores and CNV amplitude were collected and compared between conditions with and without social context. Based on the abovementioned studies, we hypothesized that: (1) on a behavioural level, participants of the social group (observed by another participant) would experience less pain and show longer reaction times in response to pain compared with participants exposed to pain without a social context; and (2) crucially, on an electrophysiological level, we expected the CNV to mimic these results, showing a decreased amplitude in the social group compared with the no-social group. Finally, since empathy may affect sensory states, including pain, and it is an essential component of the social context, empathy trait was measured, as already done in previous studies [20, 23]. Given this literature, we hypothesized empathy scores to correlate with pain ratings and reactions times only in the social group, where an observer was present.

## Materials and methods

### Participants and experimental groups

A total of 63 healthy right-handed volunteers (29 males, 34 females, age = 21 ± 2.2) were recruited among the students of the University of Turin and were engaged in the study after signing a written informed consent form. Based on our previous study all recruited participants were informed that they would take part in a study investigating pain perception in which they would receive or observe someone else receiving a train of electrical stimuli with different intensities on the dorsum of the left hand [6].

**Fig. 1:**
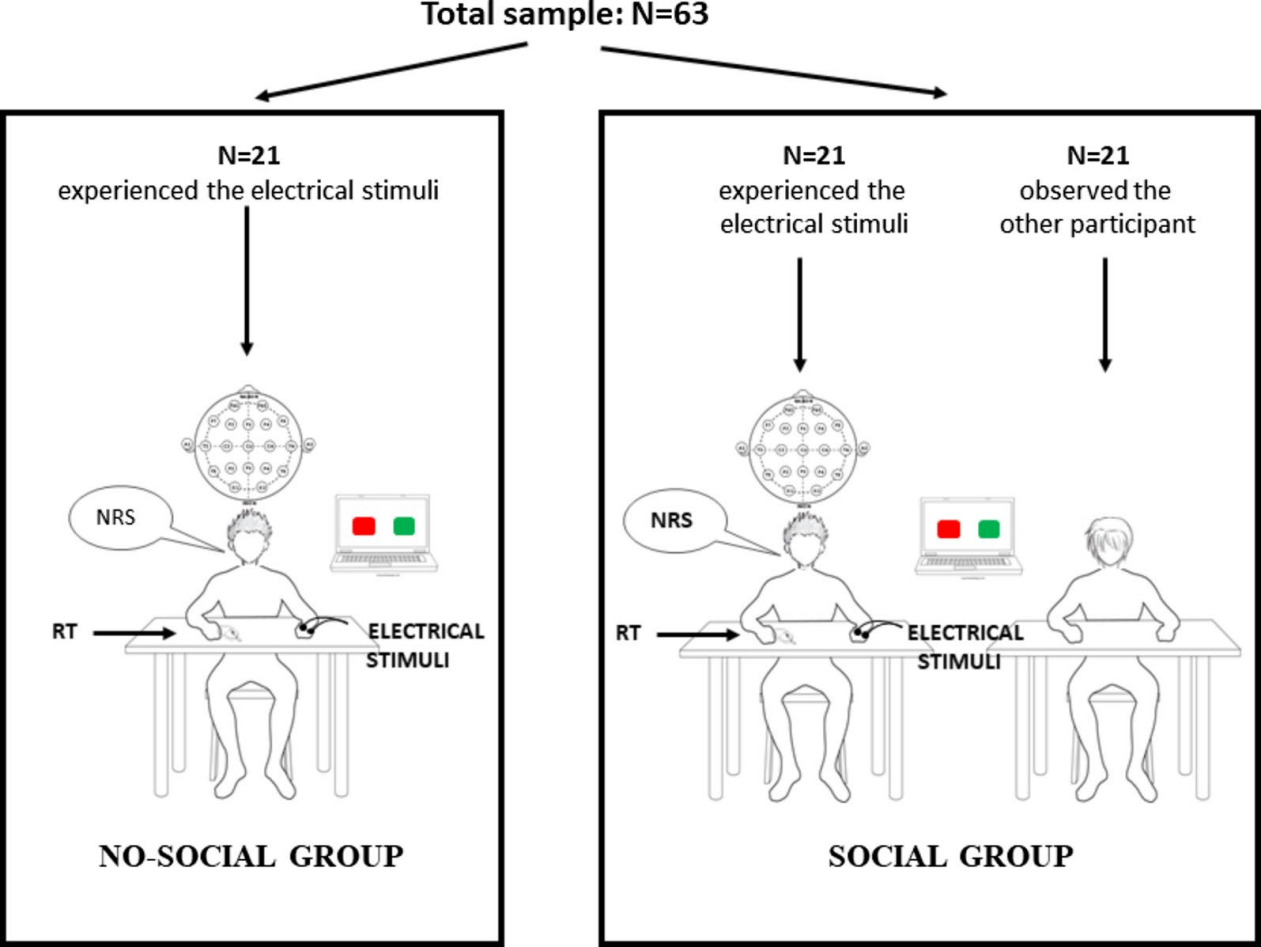
Experimental groups and set-up

Participants were randomly divided in three groups (see Fig. 1): 21 participants (10 males, 11 females, age = 20.8 ± 2.1) have been assigned to the “no-social group” and received the electrical stimuli alone (e.g., without other participants in the room), 21 participants (10 males, 11 females, age = 21.1 ± 2.4) have been assigned to the “social group” and received the electrical stimuli in the presence of an observer, the remaining 21 participants (9 males, 12 females, age = 21 ± 2.1) served as observers as they had to simply stay in the same room where participants of the social group received the stimulations. Thus, in the social group different dyads were formed: 12 dyads were “gender-matched” (5 dyads with male–male and 7 dyads with female–female), whereas 9 dyads were “gender-mixed” (4 dyads with female as observer and male as participant and 5 dyads with male as observer and female as participant). No clinical screening, pain threshold assessment or statistical analysis has been conducted on the observers, since no electrical stimuli or EEGs have been recorded.

Participants were told that when a green cue was displayed on a computer screen a low-intensity electrical stimulation would follow (low pain condition), whereas when a red cue was displayed a high-intensity electrical stimulation would follow (high pain condition). Crucially, participants of both groups were instructed to stop the electrical stimulation as fast as they could by pressing with the index finger the mouse button.

Before the experiment, participants underwent a clinical screening aimed to rule out the consumption of medications (e.g., painkillers) and caffeine beverages in the previous 12 h. Moreover, in the social group, empathy was assessed by means of the Empathy Quotient (EQ) [11]. All the experimental procedures were conducted according to the policies and ethical principles of the Declaration of Helsinki. The study was approved by the local ethics committee.

### Experimental design

The experimental design is based on a previous study on pain expectation [6]. Participants that received the electrical stimuli, sat on a chair with both hands placed on a desk. The EEG was recorded from 19 scalp locations (Fp1, Fp2, F3, Fz, F4, C3, Cz, C4, P3, Pz, P4, P7, P8, T3, T4, T7, T8, O1, O2) in accordance with the 10–20 international system (Galileo, EBNeuro, Firenze, Italy) with linked common ears reference. Impedance was less than 5 kΩ in each active lead. Data were collected and digitized at a sampling rate of 512 Hz.

After EEG electrodes placement, two electrodes were positioned on the dorsum of the left hand of participants that received the electrical stimuli and were used to deliver the train of electrical stimuli. The electrical stimuli were square pulses delivered by a somatosensory stimulator (Neuroscan, Compumedics, Charlotte, NC, USA) with a 50-µs duration and a 2-Hz frequency that lasted until the motor response. A light, visible to participants and observers, appeared on the somatosensory stimulator at each pulse, indicating that the stimulation was delivered.

The stimuli were delivered during a video presented simultaneously on two screens: a 15-in. screen approximately 1 m from the participant and a 15-in. screen approximately 1 m from the observer. The video contained the following sequence: after a 4-s asterisk indicating the fixation point, a warning cue consisting in a square (red or green) was presented in the centre of the screen for 500 ms. Then, after 3500 ms, an imperative stimulus, consisting in a train of electrical shocks to be stopped as soon as possible, was delivered. Finally, after the stimulation was stopped, a sentence asking participants to rate the stimulus (from 0 to 10) appeared. The whole sequence was created using Presentation software (Neurobehavioral System, Inc.) using a pseudorandom order, as already done in previous studies [5, 6, 23]. The intensity of the electrical shocks was based on the individual pain threshold (*T*) of each participant and on the cue. In fact, at the beginning of the experiment, right after the positioning of the skin-electrodes, *T* was assessed for each participant using the staircase method [12] and the intensity of the stimuli was set at T − 20% mA when the green cues were presented and 2*T* mA for the red cues, as already done in a previous study with the same paradigm [6]. 40 stimuli were delivered for each participant (20 low-painful stimuli associated with green cues and 20 painful stimuli associated with red cues).

After each stimulation, participants (in both the social and no-social group) were asked to stop the train of stimuli as soon as possible by pressing the left mouse button with the right hand, and their reaction time (RT) was measured. Moreover, each participant was asked to rate the intensity of the electrical stimuli using a numerical rating scale (NRS) from 0 (no pain) to 10 (maximal pain sensation).

### Behavioural analysis

Differences in the mean NRS scores as well as mean RTs were evaluated by mean of two different mixed factor 2 × 2 ANOVAs with Cue (Red vs Green) as a within factor and Group (no-social vs social) as a between factor. Finally, for the social group only, Pearson correlation coefficients were calculated between NRS scores and EQ scores and between RTs and EQ scores.

### Electrophysiological analysis

EEG continuous data were pre-processed and analysed using Matlab (Mathworks Inc., Natick, MA, USA), via the EEGLAB toolbox [24], and were synchronized to the trigger (i.e. the cues’ onset) by means of Presentation software (Neurobehavioral System, Inc). EEG data for each participant were segmented into 40 epochs (i.e. the number of trials recorded) of 7 s each (from 1 s before the warning cue to 6 s after the warning). Trials were grouped depending on the cue preceding the electrical shocks (green or red cues), and epochs preceded by the same cue were averaged together, time-locked to the onset of the cue. Each epoch was baseline corrected using the pre-warning interval from − 1 s to 0 s as reference. EEG epochs were low pass filtered below 30 Hz using fast Fourier transformation. Epochs with amplitude values exceeding ± 75 µV were rejected: a total of 1.21 ± 1.67 epochs have been removed in the social group, and 2.09 ± 1.92 in the no-social group (min epochs = 0, max epochs = 5 for both groups). Electrooculogram artefacts were subtracted through a validated method based on independent component analysis (ICA) [25].

For each participant we obtained two averages: one average corresponded to CNV after the red cue, the other to CNV after the green cue. It has been shown that the early component of the CNV usually starts after the warning cue and is more related to the sensory elaboration of the cue as well as to the meaning of the cue (i.e. expectation), while the late component starts 1 s before the imperative stimulus and is more related to the motor preparation [6, 9, 10, 26, 27]. Since the period between the warning and the imperative stimulus was more than 3 s, based on the abovementioned studies, the CNV was divided in two components, early and late. The early CNV was thus identified in a time window between 1 and 2 s after the cue onset, whereas the late CNV was identified between 3 and 4 s after the cue onset. Electrodes were grouped in three main areas based on previous studies on CNV: F3, Fz and F4 were grouped as frontal electrodes, C3, Cz and C4 as central electrodes, and P3, Pz and P4 as parietal electrodes [6, 13, 15]. Thus, three different signal averages have been created, namely frontal, central and parietal.

For each time period (early and late), differences in the area under the curve (AUC) were tested by a 2 × 3 × 2 mixed factors ANOVA, considering Cue (Red vs Green) and Area (frontal vs central vs parietal) as within factors and Group as a between factor (no-social vs social). To explore significant interactions, post hoc Student–Newman–Keuls (SNK) was applied for multiple comparisons.

For all the analyses, data in the figures are presented as mean ± standard error of the mean (SEM), and the level of significance was set at *P* < 0.05.

## Results

### Behavioural results

**Fig. 2:**
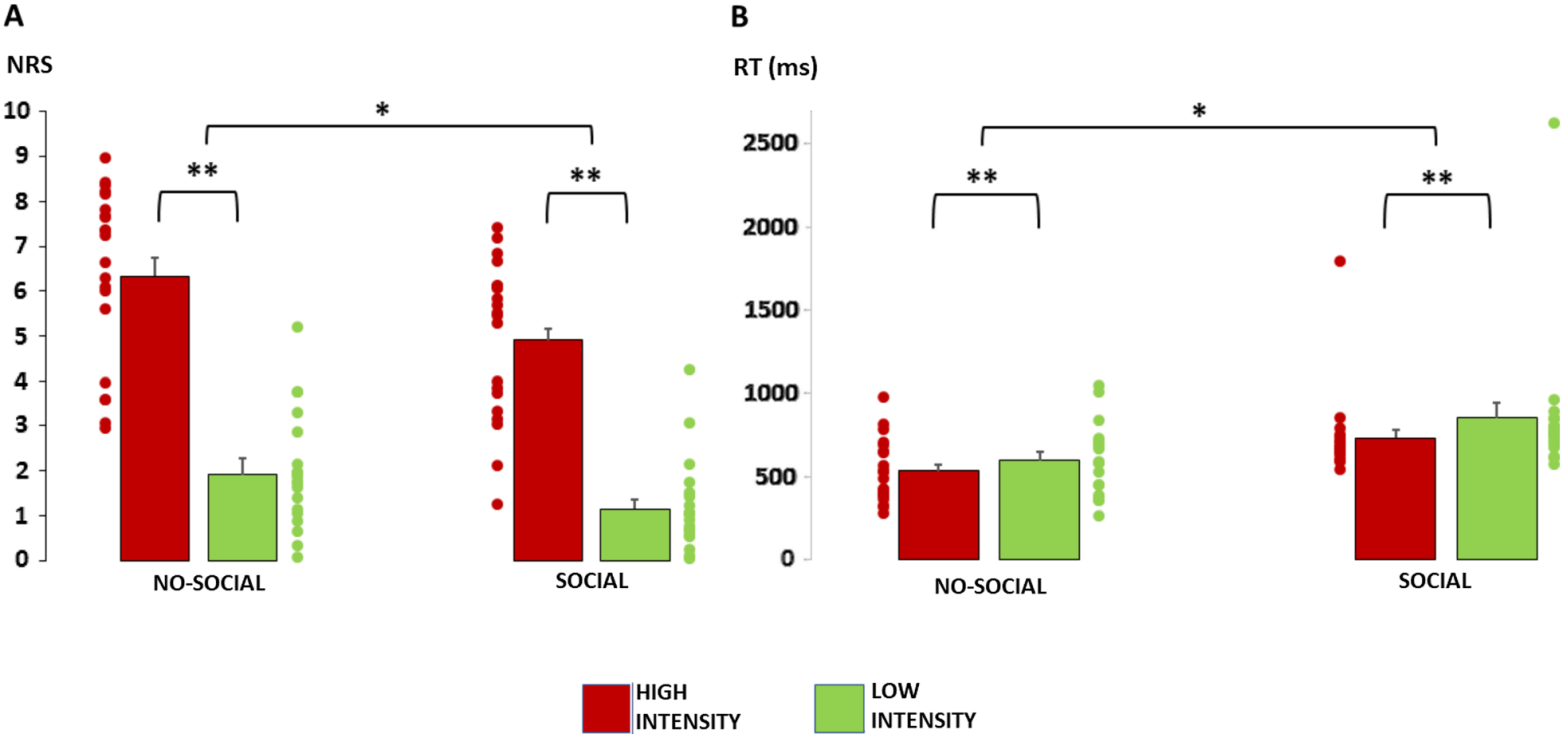
**A** On the Y-axis the NRS scores (pain perception) in the no-social and social group after the red or green cue. **B** On the Y-axis the reaction times (pain response) in ms in the no-social and social group after the red or green cue. *P < 0.05; **P < 0.01. Error bars represent standard error of the means (SEMs)

Results of the 2 × 2 ANOVA on NRS scores showed a significant main effect of Group [F(1,40) = 9.84, *P* < 0.01], showing a significantly higher pain perception for no-social in comparison to social group, and Cue [F(1,40) = 181.58, *P* < 0.001], confirming a significantly higher pain perception after the stimuli following the red cues in comparison to the stimuli following the green cues for both groups. No significant Cue X Group interaction was found [F(1,40) = 1.19, *P* > 0.05] (see Fig. 2A).

Results of the 2 × 2 ANOVA on RTs showed a significant main effect of Group [F(1,40) = 7.44, *P* < 0.01], showing a significantly longer RTs (i.e. slower responses in stopping the stimulation) in social compared to no-social group, and Cue [F(1,40) = 14.43, *P* < 0.001], confirming a significantly higher RTs after the stimuli following the green cues in comparison to the stimuli following the red cues for both groups. No significant Cue X Group interaction was found [F(1,40) = 1.38, *P* > 0.05] (see Fig. 2B).

No significant correlation was found in the social group between EQ and NRS scores, between EQ and RTs and between EQ and AUC. Furthermore, no significant correlation was found between AUC and behavioural measures.

### Electrophysiological results

**Fig. 3:**
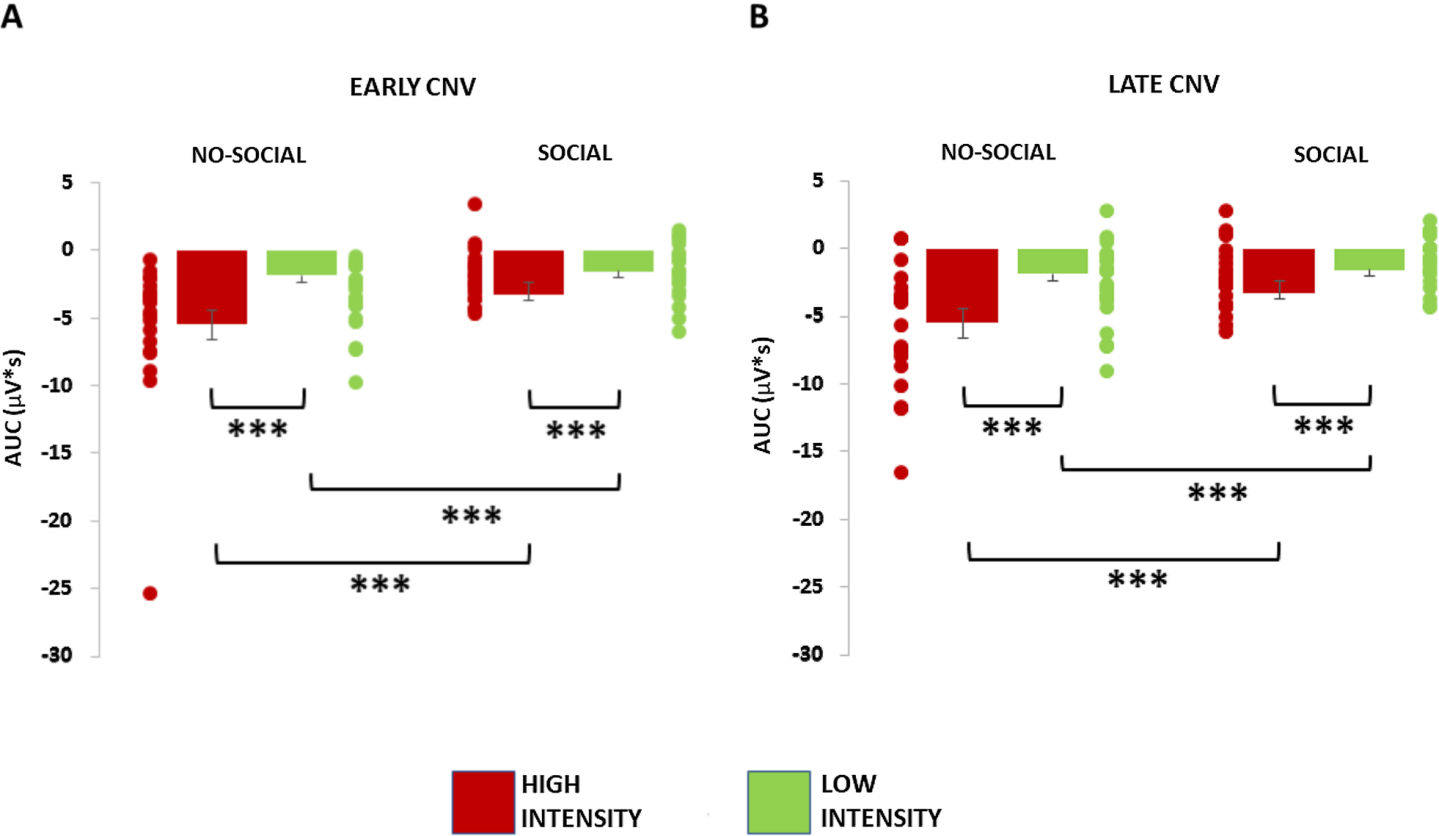
Electrophysiological results. **A** Early CNV phase. **B** Late CNV phase. On the Y-axis the AUC (microvolts*s). Direct comparison of the CNV between the no-social group and the social group. *P < 0.05; **P < 0.01; ***P < 0.001

**Fig. 4:**
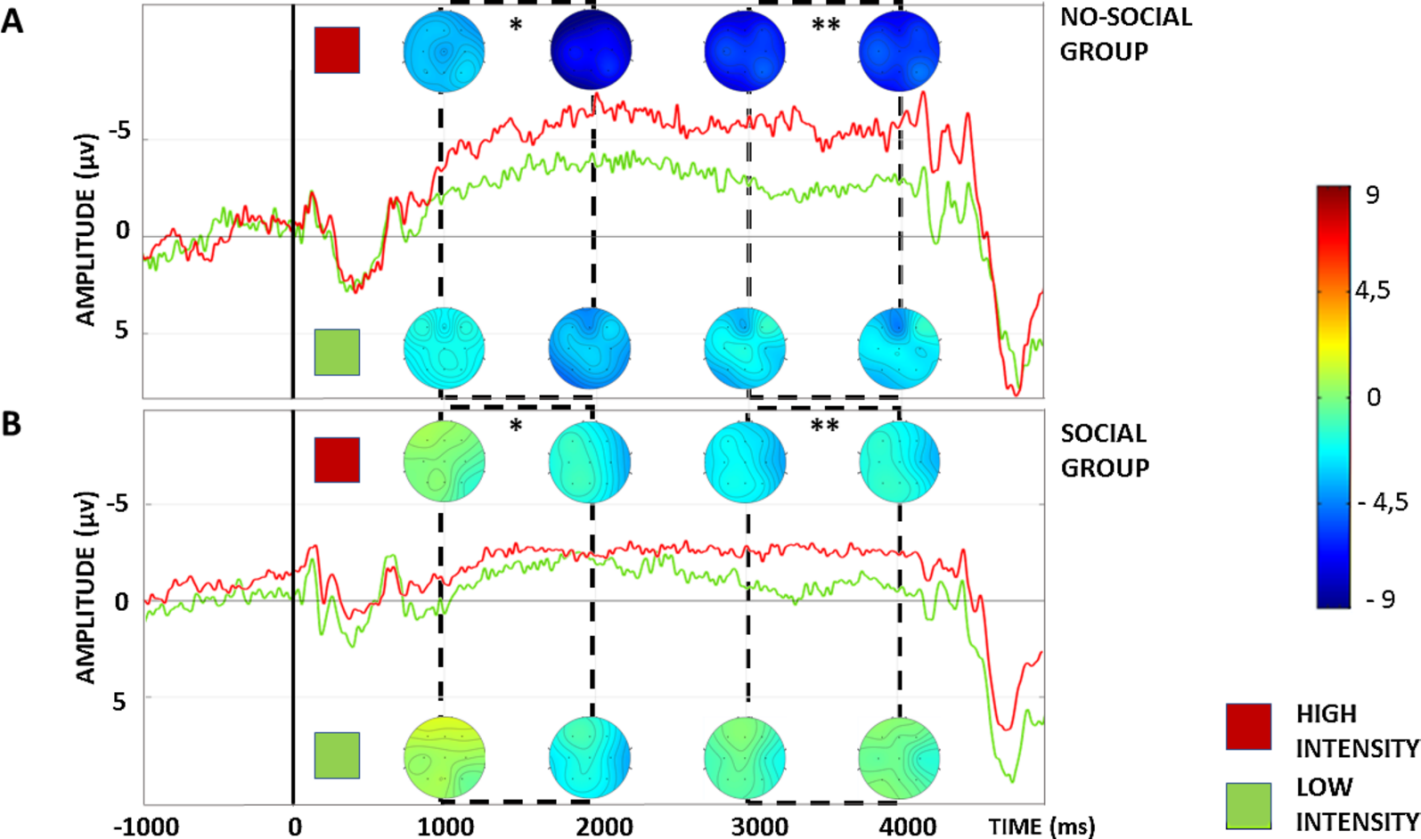
Electrophysiological results. On the Y-axis the amplitude (microvolts); on the X-axis the time (milliseconds). All CNV depicted in the figure represent the average of the nine electrodes considered (i.e. F3, FZ, F4, C3, CZ, C4, P3, PZ, P4) and dotted rectangular area represent the early (between 1000 and 2000 ms) and late (between 3000 and 4000 ms) CNV. **A** Direct comparison of the CNV after red and green cues in the no-social group. **B** Direct comparison of the CNV after red and green cues in the social group. *P < 0.05; **P < 0.01

**Fig. 5:**
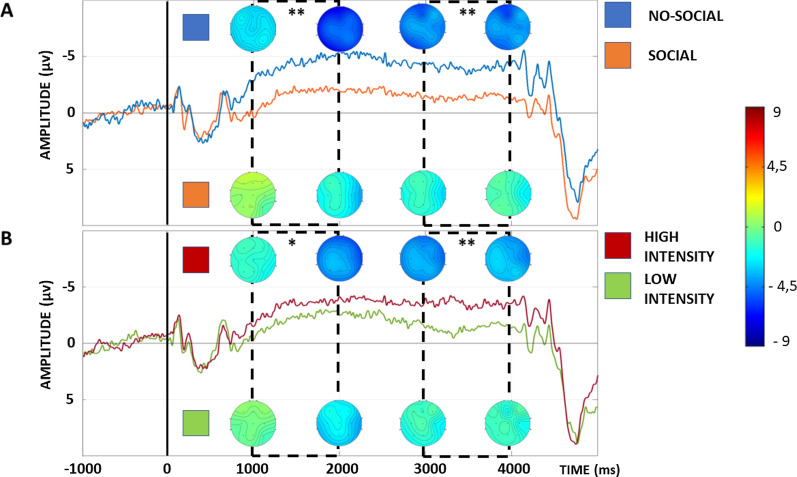
Electrophysiological results. On the Y-axis the amplitude (microvolts); on the X-axis the time (milliseconds). All CNV depicted in the figure represent the average of the nine electrodes considered (i.e. F3, FZ, F4, C3, CZ, C4, P3, PZ, P4) and dotted rectangular area represent the early (between 1000 and 2000 ms) and late (between 3000 and 4000 ms) CNV. **A** Direct comparison of the CNV between the no-social group and the social group. **B** Direct comparison of the CNV between the red and green cues for both groups. *P < 0.05; **P < 0.01

Electrophysiological results are summarized in Figs. 3, 4 and 5. The analysis on the early CNV showed a main effect of Group [F(1,40) = 10.78, *P* < 0.01] and Cue [F(1,40) = 4.87, *P* < 0.05]. In particular, the analysis showed a significantly lower early CNV amplitude (i.e. less negative) in the social group in comparison to the no-social group. Furthermore, the analysis showed a significantly higher early CNV after the red cues’ onset, leading to high-intensity stimulation, i.e. painful, in comparison to green cues, leading to low-intensity stimulation, i.e. less painful, perfectly mimicking the NRS results (see Fig. 3A). Moreover, a significant Cue x Area interaction was found [F(2,80) = 5.93, *P* < 0.01]. SNK post hoc test confirmed a significant higher early CNV after red cues in comparison to green cues for frontal (*P* < 0.01), central (*P* < 0.01) and parietal (*P* < 0.01) electrodes as well as a significant lower amplitude after the green cues in the frontal electrodes compared to the central (*P* < 0.05) and parietal (*P* < 0.01) electrodes. No other significant interactions were found.

Similarly to the early phase, the analysis on the late CNV component showed a main effect of Group [F(1,40) = 12.58, *P* < 0.01] and Cue [F(1,40) = 18.25, *P* < 0.001]. In particular, the analysis showed a significantly lower late CNV amplitude in the social group in comparison to the no-social group. Finally, the analysis showed a significantly higher late CNV after the red cues’ onset in comparison to the green cues for both groups, again mimicking the RTs results (see Fig. 3B). No other significant interactions were found (Figs. 4 and 5).

## Discussion

In this study, we sought to better understand the sensory and motor characteristics of pain perception and anticipation in a social context. In particular, we used a specific slow cortical potential, CNV, to investigate these differences. To this aim, two groups of participants were recruited: one group was instructed to expect and stop low-intensity electrical stimulations, preceded by green cues, or high-intensity electrical stimulations, preceded by red cues, without any other participant in the room (i.e. without social context), while the other group followed the same paradigm with another participant in the room (i.e. with social context).

Behavioural results highlighted that subjective pain perception, as reported by participants’ NRS scores, significantly decreases in the presence of an observer, that is adding a social context, independently of the intensity of the electrical stimuli. Furthermore, reaction times in stopping the stimulation were significantly slower in the social group.

These data are in line with different studies in which pain threshold as well as pain tolerance increased in the presence of active or passive observers [19, 28, 29]. For example, the present data are also in line with classic studies on the interpersonal influences on pain perception showing that the presence of other person increases pain tolerance, whether these observers are significant ones [28, 30] or strangers [28]. Interestingly, in a study of Edwards and collaborators, the authors found that the sex status of the dyads recruited had a significant effect on the objective measures of pain so that, for instance, the presence of male friends further increased men’s pain tolerance. However, this gender effect was only present when participants of the dyads were closely related and defined as friends [19]. Since our study was not focused on specific differences between male and female, we did not further analyse gender differences in our data. Still, future studies on central aspects of pain anticipation in social context should focus on a higher number of participants to better investigate gender and closeness characteristics. One hypothesis to explain our results is that the reduced anticipation of pain could be the product of a socially desirable behaviour also present in other animals, that is the ability to hide weakness and leave the impression of strength in others [29]. Interestingly, in a study not focused on anticipation of pain nor reaction to pain, the authors found opposite results, they explained this lack of positive effects of the social context by diming the observers as “unimportant” since they were completely unrelated to participants who received and rated painful stimuli, minimizing the social context and thus reducing its positive effect [29]. The possible difference with our study is that participants, in our case, were more engaged in the task since they had to stop as fast as they could the stimulation, which still represent a performance task, and more prone to be enhanced by the presence of observers [31]. Secondly, in our study participants and observers were selected from the same university and thus they were not completely unrelated with each other. Future studies should add psychological measures of closeness such as the relationship closeness inventory [32], to better understand if the social distance between participants and observers can modulate these positive effects of the social context.

It is worth noticing that our results could also be partially explained by attentional mechanisms. Indeed, it has been showed in different studies [33–35] that reorienting attention away from the pain stimulation and toward another sensation or scenario that is happening concurrently to the stimulation itself represents a mechanism capable of reducing pain perception. Thus, the presence of another person in the same room (i.e. the observer) could be seen as a distracting target for participants who received the electrical stimulation, resulting in lower pain perception and slower reaction times. However, it has been also found that is hard to actually shift attention from pain to other targets as they are described as less motivating for participants since they are not directly related to pain sensation which is the main focus of attention [35, 36]. To better answer this question, future studies on pain anticipation and motor reaction to pain should add experimental conditions (or groups) where the same task is performed while participants are required to focus their attention on sounds or visual stimuli presented in the same room to control for possible attentional effects.

Interestingly, our behavioural results showed reduced pain perception and slower reaction times in the social group in comparison to the no-social group for both types of stimuli, that is for both low painful as well as painful stimuli. Still, within both groups, participants felt significantly more pain after the stimuli following the red cues and consequently showed also faster reaction times to interrupt these stimuli in comparison to the stimuli following the green cues showing that these two types of stimuli were anticipated and perceived as distinctly different. One explanation for these results could be that the presence of the observer attenuated not only pain perception in participants of the social group but also their somatosensory perception. Indeed, there are different studies showing somatosensory modulation while observing another person’s body such as the modulation of tactile perception while watching another person’s hand [37] or body [38]. Still, a recent study found no evidence for somatosensory attenuation of touches following the observation of another person performing the same task [39]. Thus, further data are needed to explore this hypothesis as future studies should employ, using the same task presented in this study, somatosensory stimuli far below the pain threshold to understand if this reduced pain perception and motor reaction to pain in the presence of an observer can be found also with innocuous (i.e. non-painful) stimuli.

Furthermore, our results showed that EQ scores didn’t correlate with NRS scores or reaction times in the social group, showing no relationship between empathy and these behavioural measures during the experimental task. In a previous study focused on observational learning effects in pain mechanisms, it has been showed that participants who first observed another person feeling less pain after the administration of a (sham) analgesic cream, when later received and rated painful stimuli, presented a reduced pain perception after being administered the same (still sham) analgesic cream that they have seen previously and this reduction positively correlated with participants’ empathy scores [23]. While this result shows how empathy has a role in observational learning effects in pain mechanisms and seems in contrast with the results of the current study, it is worth noticing that in our experiment participants knew of the presence of another person in the same room but this observer did not perform any task or received any painful stimulation. Thus, one explanation for this lack of correlation between empathy and pain perception could be represented by the lack of emotional characteristics of the observer. Still, it is worth mentioning that another possible explanation for the lack of significant correlations could stem from an underpowered experimental design to due the low number of participants and future studies should focus on a bigger sample size to better investigate possible correlations.

Although electrophysiological results were not directly correlated with the behavioural data, probably due to the general high variance of EEG signals and the use of electrical and not laser-evoked pain [40], electrophysiological results still “mimicked” the behavioural results. Indeed, the early component of the CNV had a significantly lower amplitude in the social group after the onset of both cues. Similarly, the late component of the CNV showed a lower amplitude in the social group after the onset of both cues. CNV has been classically divided in two components, namely early and late CNV [9] with different neural generators as well as different roles in the expectation related to pain. The early component is generated by different areas encompassing the prefrontal cortex, the anterior cingulate cortex as well as the premotor and supplementary motor areas [13, 15] and seems to be more related to the initial attention to the warning stimulus and its meaning as well as to the cognitive processes and motivation to respond to the following imperative stimulus (i.e. in our case, the noxious stimulation) [18, 41]. In particular, in the pain domain, this early component seems to be related to the expected intensity of an incoming stimulus and its subjective pain perception [6]. Indeed, the lower early amplitude of the CNV observed in the social group is completely in line with the NRS results in this group showing a significantly lower pain perception after the stimulation following both cues.

In contrast with the early component, the late component of the CNV is observed before the onset of the imperative stimulus and is originated mainly by motor areas such as the basal ganglia, the premotor cortices but also the dorsal anterior cingulate cortex [13, 15, 16], being clearly related to motor preparation [17, 42]. In a paradigm where the imperative stimulus is represented by a train of painful stimuli, it has been previously observed how the amplitude of the late CNV is related to the reaction times involved in stopping the stimulation [6]. Indeed, motor preparation seems to be a crucial part of pain anticipation and perception as pain itself can be conceived not only as a signal of a harmful stimulus but also as a signal to start a specific action. Again, in our study, results showing a lower amplitude of the late CNV in the social group after the red as well as the green cues are in line with the behavioural results showing slower reaction times in the social group in stopping the stimulation after both the red and green cues. As a side note, early CNV measured in the frontal electrodes after the presentation of green cues showed a significantly lower amplitude in comparison to central and parietal electrodes. This result could be possibly explained because frontal areas are less involved in the generation of this early component, while being more involved with the motor part of the CNV, as previously described. Furthermore, the green cue represented the stimulus after which the lower intensity stimulation was administered and, thus, the slower motor response, for both groups, was observed. This combination of information, i.e. early component as well as the expectation of a low intensity, could have explained why the lowest CNV amplitude was observed in the frontal electrodes after the green cue.

## Conclusion

Data from the current study further expand the previous results highlighting that CNV can be a useful tool to dissect sensory expectation and motor preparation in response to pain in a social context. While all the acquired data depict a clear picture of the electrophysiology behind pain anticipation in a social context, some limitations need to be considered. One of the main focuses of this study was to better characterize the central elaboration of pain anticipation and motor preparation in a social context using a specific evoked potential, that is the CNV. Future studies on this topic should use high-density EEG to better investigate possible source differences of the CNV between social and non-social contexts. Furthermore, a higher number of participants is necessary to better analyse possible gender differences.

## Data Availability

The datasets used and analysed during the current study are available from the corresponding author on reasonable request.
